# Can virtual nature improve patient experiences and memories of dental treatment? A study protocol for a randomized controlled trial

**DOI:** 10.1186/1745-6215-15-90

**Published:** 2014-03-22

**Authors:** Karin Tanja-Dijkstra, Sabine Pahl, Mathew P White, Jackie Andrade, Jon May, Robert J Stone, Malcolm Bruce, Ian Mills, Melissa Auvray, Rhys Gabe, David R Moles

**Affiliations:** 1 School of Psychology, Cognition Institute, Plymouth University, Plymouth, UK; 2 European Centre for Environment and Human Health, University of Exeter Medical School, Knowledge Spa, Royal Cornwall Hospital, Truro, Cornwall TR1 3HD, UK; 3 University of Birmingham, Birmingham, UK; 4 Plymouth University Peninsula Schools of Medicine and Dentistry, Plymouth, UK; 5 Torrington Dental Practice, Torrington, UK

**Keywords:** Dental anxiety, distraction, memories, virtual reality

## Abstract

**Background:**

Dental anxiety and anxiety-related avoidance of dental care create significant problems for patients and the dental profession. Distraction interventions are used in daily medical practice to help patients cope with unpleasant procedures. There is evidence that exposure to natural scenery is beneficial for patients and that the use of virtual reality (VR) distraction is more effective than other distraction interventions, such as watching television. The main aim of this randomized controlled trial is to determine whether the use of VR during dental treatment can improve the overall dental experience and recollections of treatment for patients, breaking the negative cycle of memories of anxiety leading to further anxiety, and avoidance of future dental appointments. Additionally, the aim is to test whether VR benefits dental patients with all levels of dental anxiety or whether it could be especially beneficial for patients suffering from higher levels of dental anxiety. The third aim is to test whether the content of the VR distraction can make a difference for its effectiveness by comparing two types of virtual environments, a natural environment and an urban environment.

**Methods/design:**

The effectiveness of VR distraction will be examined in patients 18 years or older who are scheduled to undergo dental treatment for fillings and/or extractions, with a maximum length of 30 minutes. Patients will be randomly allocated into one of three groups. The first group will be exposed to a VR of a natural environment. The second group will be exposed to a VR of an urban environment. A third group consists of patients who receive standard care (control group). Primary outcomes relate to patients’ memories of the dental treatment one week after treatment: (a) remembered pain, (b) intrusive thoughts and (c) vividness of memories. Other measures of interest are the dental experience, the treatment experience and the VR experience.

**Trial registration:**

Current Controlled Trials ISRCTN41442806

## Background

Results from the latest National Health Service (NHS) Adult Dental Health Survey showed that over a third of adults (36%) suffer from moderate dental anxiety and another 12 percent from extreme dental anxiety [1]. Managing dental anxiety is recognized as an important issue in dental practice [2], since many people avoid or delay dental care because they experience fear and anxiety [3], and the expectation of pain is seen as a major barrier to seeking dental care [4]. Patients with dental fear tend only go to the dentist when they experience pain, thereby increasing the chance that their visit to the dentist will involve pain and exacerbating their anxiety [5]. Dentists themselves suffer from heightened discomfort when treating anxious patients [6]. Alleviating dental anxiety may also reduce perceived pain and unpleasantness [7], which may facilitate the work of dentists [2]. Dental anxiety and anxiety-related avoidance of dental care thus create a significant problem for both patients and the dental profession.

Previous poor medical experiences have been associated with anxiety and pain [8] and this may negatively impact the likelihood of patients complying with the required frequency of dental appointments. Moreover, anxious patients are less likely to keep their appointments [9], take longer to treat when they do attend, and afterwards feel less satisfied with their treatment [10]. Armfield and colleagues [5] provided evidence for the existence of a vicious cycle of dental anxiety. They demonstrated that people with high dental fear are more likely to delay dental treatment, which leads to more extensive dental problems and symptomatic visiting patterns, which in turn feed back into the maintenance or exacerbation of existing dental fear. Moreover, dental anxiety is associated with the tendency to experience negative or threatening thoughts concerning treatment [11].

Heightened emotion and arousal during an event increases the likelihood of recollections of the event being triggered uncontrollably by situational cues [12], for example, a whiff of antiseptic that triggers thoughts about dental treatment. Attempts at suppressing these intrusive thoughts tend to be counterproductive [13]. Once triggered, intrusive thoughts tend to be elaborated [14]. For example, an intrusive thought about going to the dentist might lead to the patient imagining how uncomfortable the next visit is going to be. According to the elaborated intrusion theory [14], the imagined experience not only simulates the sights and sounds of the experience but incorporates the actual emotions associated with that experience. This project aims to break this cycle of dental anxiety by providing pleasant imagery during dental treatment that distracts patients from treatment. We hypothesize that this imagery will block the development of intrusive memories of the dental experience, and render voluntary memories of the experience less vivid and less emotive; in particular, we expect remembered pain to be less intense.

There is a growing body of research that provides evidence that providing positive distractors in healthcare environments can impact health-related outcomes, such as anxiety and well-being of patients (for reviews, see [15,16]). Another review concluded that viewing nature scenes might decrease pain perceptions by eliciting positive emotional responses and decreasing stress levels [17]. Research on restorative environments suggests that certain environments, especially natural settings [18], are better at promoting recovery from stress. Lohr and Pearson-Mims [19] studied whether the presence of indoor plants would increase pain tolerance. Participants were placed in either a room with plants, a room with non-plant objects (as visually distracting as the plants), or a control room (no objects). A larger proportion of respondents in the room with plants were able to keep their hand in iced water for 5 minutes as compared with the participants experiencing the other conditions, suggesting increased pain tolerance by exposure to indoor plants. Both studies introducing real nature and studies using artificial nature interventions (for example, posters) show positive effects on patient outcomes [20-22]. Using nature to provide distraction in the dental clinic might thus lead to beneficial effects on the patient experience.

A variety of distraction interventions are used in daily medical practice to help patients cope with unpleasant procedures; these include watching television and listening to music [23,24]. We selected virtual reality (VR) as a vehicle for presenting nature to patients during dental treatment, based on evidence from a case study in dentistry showing that exposure to VR distraction is more effective in offering pain control than exposure to a video or a standard care situation without distraction [25].

Virtual reality goggles not only display potentially attractive visual stimuli to look at, they also exclude all other visual stimuli in the environment that might affect the patient. It has been suggested that, in medical surgical settings, the appearance of a nurse who cleans patient’s wounds can be strong enough to create anxiety [26]. The overhead light, the dentist with a facemask and the dental instruments might induce anxiety in a similar way. Virtual reality goggles effectively exclude the treatment environment and are thus a potentially interesting way of offering distraction to patients. Another reason why VR can be more effective than other distraction interventions is the difficulties some people may have in evoking images that are vivid enough to be effective distractors [27]. Additionally, using VR can offer a feeling of control. Previous research for example demonstrated that interactive VR, which offered more control possibilities, was better than passive exposure in children [28,29].

Several small-scale studies explored the use of VR in a dental context. A first study investigated the effects of using an audiovisual eyeglass system that displayed an instructional video [30]. Adult patients scheduled for dental prophylaxis were distracted during half of their treatment. At the end of the treatment, they were asked to compare the situation with and without the VR exposure. Patients reported less anxiety and discomfort when using the equipment. In another study, patients undergoing periodontal scaling and root planing procedures were exposed to a control situation (only wearing the headgear), a video (that is, the animation movie *Cars*) and a VR environment (of a botanical garden in *Second Life*) [31]. Both distracters, relative to the control condition, resulted in less pain and discomfort and lower blood pressure and pulse rate. Moreover, the VR environment scored significantly better on all indicators than the movie.

A range of VR environments has been studied, but a systematic approach to researching what type of environment might be more beneficial has not been taken before. Previous VR research looked at forests [27], a botanical garden [31] and snowy canyons [32,33] as well as a variety of video games [28,29,34]. Although natural and game-based distracters showed benefits, little is known about what sort of VR content is most effective at reducing stress and creating positive experiences and memories of treatment. Based on the evidence [18-22] that natural settings are more restorative than built environments, this research will compare a natural and an urban virtual environment, to test not only whether VR distraction is effective, but also to explore whether the content of the VR can make a difference.

### Aims and hypotheses

The main aim of the project is to determine whether the use of virtual nature during dental treatment can improve recollections of treatment and the overall dental experience. Additionally, we will look at the moderating role of patients’ baseline level of dental anxiety in these effects. A third aim is to test whether the content of the VR distraction can make a difference for its effectiveness by comparing two types of virtual environment, a natural environment and an urban environment.

#### *Objectives*

1. Determine the effectiveness of VR in improving recollections of treatment and the overall dental experience.

2. Determine the role of patients’ level of dental anxiety and test whether the use of VR could be especially beneficial for patients suffering from dental anxiety.

3. Determine any effect relating to the content of the VR.

#### *Overall hypotheses:*

1. Virtual reality exposure will lead to less intrusive thoughts, less vivid memories, less remembered pain and better overall dental experiences.

2. Dental anxiety will moderate the proposed effects under hypothesis 1. Patients with more dental anxiety will benefit more from the use of VR than patients with less dental anxiety.

3. The natural VR environment will be more effective than the urban VR environment.

#### *Additional predictions*

##### 

**Treatment experience** Compared with standard care, VR exposure (in general) will lead to less self-reported: (1) pain, (2) discomfort and (3) stress. It will also lead to (4) a lower average heart rate (an index of physiological stress) and (5) an underestimation of time elapsed (reflecting the ability of the VR environment to engage patients). For all of these outcomes, we further predicted that the natural VR environment will produce more positive outcomes than the urban VR environment. Additionally, we predict that (6) the use of VR will lead to more perceived control.

##### 

**Dental experience** Patients exposed to VR environments will show (7) lower subsequent levels of dental anxiety than those exposed to standard care and (8) improved intentions to revisit the surgery. Again we hypothesized that these effects would be greater in the natural than in the urban VR.

##### 

**Virtual reality experience** The natural VR environment will be perceived as more (9) attractive and (10) psychologically restorative than the urban environment. Moreover, (11) VR exposure will lead to a reduced awareness of the surrounding environment.

## Methods/design

### Ethics

This study has been approved by the NRES Committee West Midlands, Coventry and Warwickshire (REC Reference 13/WM/0152) and registered with Current Controlled Trials (registration number: ISRCTN41442806).

### Intervention

**Figure 1:**
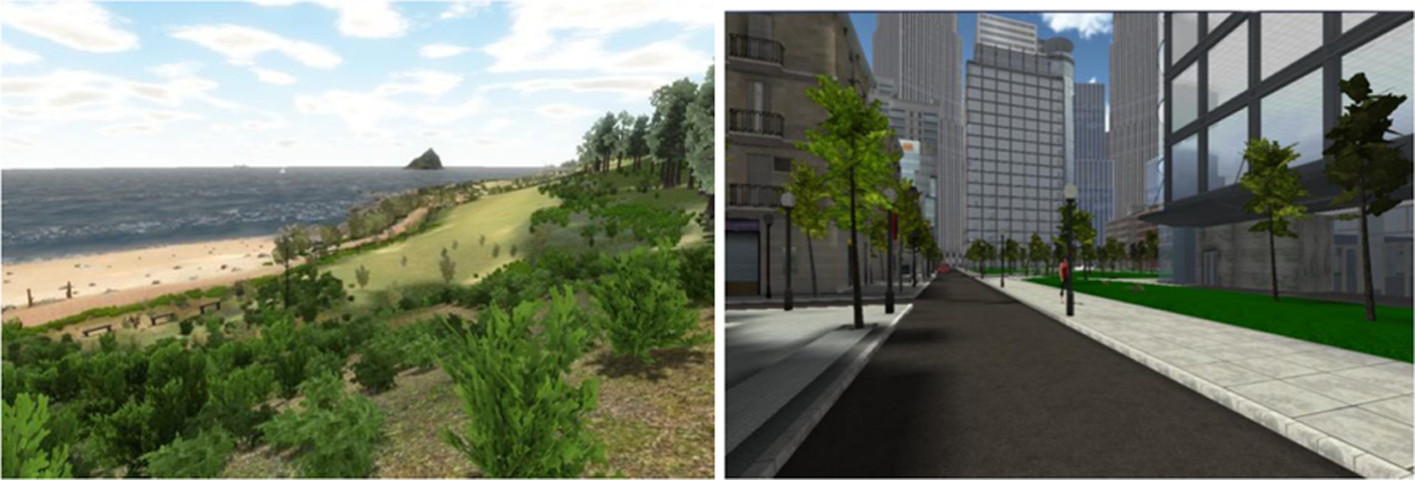
The two VR environments.

Two VR environments will be investigated in this study (Figure 1). The first VR environment consists of a simulated natural environment. The second VR environment consists of a simulated urban environment. In both groups, patients will be wearing VR goggles and will use a one-handed controller to navigate the environment.

**Figure 2:**
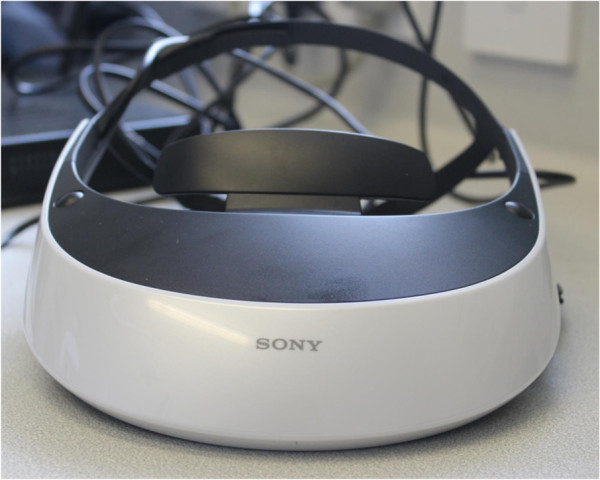
The head-mounted device (HMD).

**Figure 3:**
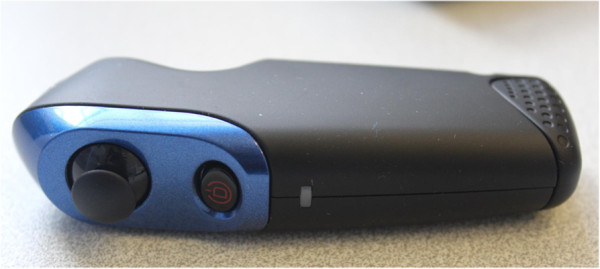
The Zeemote JS1 thumb stick controller.

A Sony personal 3D viewer headset (Figure 2) will be connected to an Alienware gaming laptop and used to display the VR environment. Participants can walk around in the virtual environment by using a Zeemote JS1 thumb stick controller (Figure 3). This controller is also used to look around, since head tracking of the head-mounted device (HMD) will be switched off.

### Study participants

Eligibility for this study is determined using the following criteria:

#### *Inclusion criteria*

1. All patients who are scheduled to undergo dental treatment for fillings or extractions with a planned maximum treatment length of 30 minutes are eligible to participate in the study.

2. This includes both referred patients and in-house patients.

3. Both patients who need relative analgesia (RA) and patients who are treated without sedation will be included.

4. Patients 18 years or older will be included.

#### *Exclusion criteria*

1. Patients who need intravenous sedation cannot participate in the study.

2. Patients who have previously had epileptic seizures cannot participate in the study, owing to the very small risk that an episode can be triggered by using the HMD, similar to the episodes that might be triggered by watching television.

3. If a patient is scheduled for more than one treatment during the trial period, the patient will only be included for the first treatment.

### Randomization process

Patients will be allocated to one of the three conditions by computer randomization using the ralloc command in the STATA software package. Randomization will be stratified by sedation (RA; yes or no) and the dentist who performs the treatment (dentist 1 or dentist 2). Randomization will be concealed via sequentially numbered opaque sealed envelopes and will be revealed by the dentist or nurse who will start the VR, immediately prior to commencing the treatment.

### Sample size

Given the novel approach of this study, there is little comparable research available to use for a sample size calculation. We are also somewhat restricted by the number of potential patients available within a given time period in the dental practice. However, we do have data from our own pilot work in a simulated dental setting, which we have used to make an informed estimate of the required sample size for the current study.

The sample size calculation (with a power of 0.80, significance level of 0.05 and based on 2-sided testing) is based on one of the main outcomes related to memories, ‘intrusive thoughts’, as measured with an 11-point verbal rating scale. In our pilot study, the standard deviation for this outcome in our control group was 1.25 and we have conservatively used an estimated standard deviation of 1.3 in this sample size calculation. Based on these assumptions, and comparing the two VR groups against the control group, following up a total sample size of 90 patients (that is, 30 per group) would allow the detection of a between-group difference of around 0.82 units (that is, a moderate effect size). Since drop-outs are unavoidable when collecting follow-up data, this number needs to be adjusted for the estimated drop-out rate. In our simulation study, we had a drop-out rate of 10% for collecting the follow-up data, but since we paid participants for participation in that study (although they received their pay after the simulated treatment and before participating in the follow-up interview), we anticipate a higher drop-out rate for this study, and have allowed for a 15% drop-out rate. Adjusting the sample size for this drop-out rate results in a sample of 108 patients needing to be recruited.

The use of technical computer equipment is associated with the risk of technical failures. In the pilot study we had to exclude 6 of 75 participants because the VR crashed or the remote stopped working during the simulated treatment and the participant was no longer able to interact with the VR environment. Several improvements in the VR program have been made since, such as putting force fields in place that restrict the area of virtual exploration, but we still anticipate the exclusion of participants because of technical issues. We will therefore increase the sample size by a further 8%, resulting in a total recruitment target of 120 for this study (that is, 40 patients per group).

### Procedure

This study will include both referred patients and in-house patients from a dental clinic in the United Kingdom. Since there are some differences in the timing and scheduling of contacts between the practice and the two types of patient, two separate procedures are described.

#### *Referral patients*

Referral patients will receive a letter from the practice about their appointment date in the post. The patient information leaflet about the study will be included. On average, this is about 12 weeks before the treatment takes place.

When the patient comes in for a preoperative assessment, informed consent will be obtained, at the end of this appointment. On average, this is 4 weeks before the treatment appointment. After obtaining informed consent, and at the end of the preoperative assessment appointment, the dentist or nurse will hand a baseline questionnaire to the patient. The patient will be asked to complete these questions at the practice before going home. Patients will be asked to complete a dental anxiety questionnaire, reporting their oral health status and anticipated pain. Completing the questionnaire will take between 5 and 10 minutes.

#### *In-house patients*

In-house patients will have routine check-up appointments at the practice and, if they need treatment, they will be booked in for a treatment appointment. At this point, they will receive the patient information leaflet and will be asked for their informed consent. Owing to the way the healthcare process is organized in the practice, a cooling-off period at this time for the in-house patients cannot be provided. However, at this stage of the study, they will only be asked to complete a baseline questionnaire. Patients will be asked to confirm their consent when they attend their treatment appointment, which is on average 25 days later. This allows the patient to think about their participation in the trial and ask any questions they might have.

After obtaining informed consent, and at the end of the appointment, the dentist or nurse will hand a baseline questionnaire to the patient. The patient will be asked to complete these questions at the practice before going home. Patients will be asked to complete a dental anxiety questionnaire, reporting their oral health status and anticipated pain. Completing the questionnaire will take between 5 and 10 minutes.

#### *For both patient groups*

Patients will be randomized when they attend their scheduled treatment appointment (that is, after baseline data has been recorded). If a patient decides that he or she no longer wants to participate in the study, the baseline data will be discarded. When undergoing their scheduled dental treatment, patients will be exposed to either one of the VR conditions (natural or urban VR environment) or the control condition (usual care). Standard clinical procedures will be followed and when treatment will be started, patients will either be handed a pair of VR goggles (when assigned to the VR groups) or a pair of protective glasses (when assigned to the standard care group). Patients in the VR groups will receive an explanation of how to use the controller by a member of staff. Heart rate will be monitored during dental treatment by a staff member using a pulse oximeter on a finger of the hand that is not operating the controller. The output of the pulse oximeter throughout the treatment session will be recorded by the dentist or nurse. After treatment, staff will record the treatment characteristics and patient characteristics. The patient will be asked to complete a questionnaire in the waiting room before leaving the dental practice. This questionnaire takes about 10 minutes to complete and consists of four parts; treatment experience, VR experience, dental experience and demographic information. The patient will be provided with an envelope, in which to seal the questionnaire.

One week after treatment, patients will receive a phone call, during which we will ask them about their memories and dental anxiety. This phone call will be made by one of the members of the research team and will take between 5 and 10 minutes. After responding to the questions, patients will be debriefed and will be asked if they would like to receive the results of the study. If patients ask questions regarding their clinical care during this interview, it will be tactfully explained to them that the interviewer is not a clinician and they will be directed to the practice for a response to any such queries.

### Outcome measures

#### *Main study parameters*

We developed a questionnaire that assessed intrusive thoughts of the experience and vividness of memories of the experience. This questionnaire is based on the Alcohol Craving Experience Questionnaire [35]. This was developed to assess craving based on the elaborated intrusion theory and measures vividness of memories and intrusive thoughts. The questions to measure memories and intrusive thoughts were used in our pilot work and showed sufficient reliability in that study (Cronbach’s alpha of respectively 0.69 and 0.81). They are slightly adapted to fit the purposes of the current study.

• Vividness of memories will be measured with five items on an 11-point verbal rating scale (VRS).

• Intrusive thoughts about the experience will be measured with three items on an 11-point VRS.

• Remembered pain will be measured with an 11-point VRS [36].

• Patients will respond to an open-ended question about the three things they remember most and to indicate, for each of these three things, how pleasant or unpleasant their thoughts were. They will also be asked how well they remember what the dentist said.

#### Secondary study parameters

The patient will complete questionnaires after their preoperative assessment, immediately after their treatment and 1 week after their treatment (phone call). Table 1 gives an overview of the measures, and at which time points they will be completed. The following parameters will be measured:

**Table 1:** Overview and timing of measurements

**Measures**	**Times**				**Completed by**	**Using**	**Where**
	**After preoperative assessment**	**During treatment**	**After treatment**	**Follow-up**			
**Dental anxiety**	X (T1)		X (T2)	X (T3)	Patient	Questionnaire after preoperative assessment (T1)	At the practice (waiting room) (T1 and T2)
						Questionnaire for the patient after treatment (T2)	Via phone (T3)
						Follow-up questionnaire (T3)	
**Treatment characteristics**			X		Staff	Data recording sheet for staff	At the practice
**Patient characteristics**			X		Staff	Data recording sheet for staff	At the practice
**Self-reported oral health status**	X				Patient	Questionnaire after preoperative assessment	At the practice (waiting room)
**Anticipated pain and anxiety**	X				Patient	Questionnaire after preoperative assessment	At the practice (waiting room)
**Demographics**			X		Patient	Questionnaire for the patient after treatment	At the practice (waiting room)
**Previous cancellations**			X				
**Treatment experience**			X		Patient	Questionnaire for the patient after treatment	At the practice (waiting room)
**Stress (physiological)**		X			Staff	Pulse oximeter	In treatment area
**Virtual reality experience**			X		Patient	Questionnaire for the patient after treatment	At the practice (waiting room)
**Dental experience**			X		Patient	Questionnaire for the patient after treatment	At the practice (waiting room)
**Memories (follow-up)**				X	Patient	Follow-up questionnaire	Via phone

##### Treatment experience

• Pain will be measured with an 11-point VRS [36].

• Discomfort will be measured with two questions on an 11-point VRS [25].

• Stress will be measured with a self-reported measure and a physiological indicator (heart rate). The self-reported measure consists of five items from the tension dimension from the Profile of Mood States [37]. Heart rate will be monitored using a pulse oximeter and the output will be recorded.

• Time perception will be measured with one question in which patients are asked to estimate how many minutes they think they have been wearing the VR goggles or protective glasses [38].

• Perceived control will be measured with an 11-point VRS.

##### Dental experience

• To measure communication with the dentist, patients will be asked to indicate on an 11-point VRS how much attention they paid to what the dentist said.

• Revisit intentions and likeliness of avoidance will be measured by asking participants how much they would like to avoid similar treatment in the future.

• Dental anxiety will be measured immediately after treatment and a week later at follow-up, using the Modified Dental Anxiety Scale (MDAS), which consists of five items [39].

• Treatment satisfaction will be measured with six statements, on an 11-point VRS, about participants’ last visit to a dental practice, based on the Service Quality (SERVQUAL) questionnaire [40].

##### 

**VR experience** The questions about the VR experience will only be asked to patients in the two VR groups.

• Presence will be measured with six items on 11-point verbal rating scales, based on the IGroup Presence Questionnaire [41] and the Reality Judgment and Presence Questionnaire [42].

• Perceived restoration will be measured with eight items on a 5-point scale [43].

• Attractiveness will be measured with five items on a 5-point bipolar adjective scale [19].

• Awareness of the surrounding dental environment will be measured with one question on an 11-point VRS.

• Nausea will be measured with an 11-point VRS [25].

• Intention to use VR goggles again will be measured with an 11-point VRS.

#### *Other study parameters*

Effect modifiers and possible confounders are:

##### Patient characteristics

• Dental anxiety will be measured with the MDAS [39], which consists of five items and the Monitoring Blunting Dental Scale [44].

• Anticipated pain will be measured after the patients’ preoperative assessment. This will be measured with an 11-point VRS [36].

• Anticipated anxiety will be measured after the patients’ preoperative assessment. This will be measured with an 11-point VRS [45].

• Whether the patient is funded by the NHS or privately.

• Whether the patient is a referral patient or a regular (in-house) patient.

• Self-reported oral health status will be measured with one question after the patients’ preoperative assessment [46].

• Demographic characteristics will be completed as part of the questionnaire that patients complete after treatment. Data on age, sex and education will be collected.

• Previous cancellations will be measured with one question (self-reported).

##### Treatment characteristics

• The type of treatment

• The length of treatment

• The treating dentist

• Sedation

### Data analysis

The data will be recorded and analyzed using SPSS 20.0. All data will be analyzed using an intention-to-treat analysis. Descriptive statistics will be calculated. Discrete variables will be summarized by frequencies or proportions. Continuous variables will be reported as means and standard errors or medians and range (depending on the distribution of the variables). Data will be checked for baseline differences between the treatment arms. If baseline differences do occur for any of the variables, they will be added to subsequent models to compensate for those differences using an analysis of covariance approach.

Analyses of covariance (ANCOVAs) will be performed for each of the outcome measures with VR condition (that is, natural, urban or control) as the independent variable and baseline dental anxiety as the covariate, including planned contrasts to explore specific hypotheses. Contrasts for VR condition will be based on comparisons of VR (both natural and urban environments combined) with the no VR control group (comparison 1), natural VR with urban VR (comparison 2), natural VR with the control group and urban VR with the control group (Table 2 gives an overview of specific predictions being tested).

**Table 2:** Overview of the specific comparisons for each prediction

	**Prediction**	**Comparison 1:**	**Comparison 2:**	**Comparison 3:**
		**virtual reality (both natural and urban environments) vs no virtual reality (control group)**	**natural virtual reality vs urban virtual reality**	**(a) natural virtual reality vs control**
				**(b) urban virtual reality vs control**
Overall hypotheses	1	✓		
	2	✓		
	3		✓	✓
Additional predictions	1	✓	✓	✓
	2	✓	✓	✓
	3	✓	✓	✓
	4	✓	✓	✓
	5	✓	✓	✓
	6	✓		
	7	✓	✓	✓
	8	✓	✓	✓
	9		✓	
	10		✓	
	11	✓		

## Trial status

At the time of submission of this protocol (September 2013), enrolment into the study was ongoing and so far two patients had been randomized.

## Abbreviations

ANCOVA: analysis of covariance; HMD: head-mounted device; MDAS: Modified Dental Anxiety Scale; NHS: National Health Service; RA: relative analgesia; SERVQUAL: Service Quality; VR: virtual reality; VRS: verbal rating scale.

## Competing interests

The authors declare that they have no competing interests.

## Authors’ contributions

KTD, SP and MPW contributed to the conception and design of the study and to writing the manuscript writing. JA, JM and DRM contributed to the conception and design of the study and provided critical revision of the manuscript. RJS, IM, MA and RG contributed to the design of the work and provided critical revision of the manuscript. MB contributed to the conception of the work and provided critical revision of the manuscript. All authors read and approved the final manuscript.
